# Lewis y antigen promotes p27 degradation by regulating ubiquitin-proteasome activity

**DOI:** 10.18632/oncotarget.22617

**Published:** 2017-11-08

**Authors:** Mingbo Cai, Shan Jin, Lu Deng, Liancheng Zhu, Zhenhua Hu, Dawo Liu, Juanjuan Liu, Mingzi Tan, Jian Gao, Huimin Wang, Bei Lin

**Affiliations:** ^1^ Department of Obstetrics and Gynecology, Shengjing Hospital Affiliated to China Medical University, Heping District, Shenyang 110004, Liaoning, China; ^2^ Department of Obstetrics and Gynecology, The First Affiliated Hospital of Zhengzhou University, Zhongyuan District, Zhengzhou 450000, Henan, China

**Keywords:** Lewis y antigen, ovarian cancer, p27, autophagy, ubiquitin-proteasome pathway

## Abstract

As a tumor-associated carbohydrate antigen, elevated expression of Lewis y promotes the malignant behaviors of tumor cells. Although our preliminary study showed that the increased expression of Lewis y antigen decreased the expression of cell cycle inhibitor protein p27, the relevant mechanism remains unclear. Autophagy and the ubiquitin-proteasome system are two main ways of intracellular protein degradation, whose abnormal activities are closely associated with progression of malignant tumors. In our present study, we constructed two stable transfected cell lines with high expression of Lewis y antigen, named CAOV3-FUT1 and SKOV3-FUT1. We showed that the proportion of cells at S phase was significantly increased after FUT1 transfection, whereas p27 protein was obviously decreased. The autophagy activity, the levels of ubiquitination, and chymotrypsin-like protease activity were increased remarkably in the transfected cells. Interestingly, Lewis y antigen promoted the degradation of p27 by increasing ubiquitin-proteasome activity. In the vivo studies, Lewis y antigen improved the tumorigenic ability of ovarian cancer cells in nude mice and reduced the expression of p27. These findings suggested that Lewis y antigen activated both the autophagy and ubiquitin-proteasome activity and promoted the degradation of p27 through the ubiquitin-proteasome pathway.

## INTRODUCTION

More than half of the proteins in the human body are glycoproteins. Glycosylation is one of the most important post-translational modifications of proteins, and participates in a variety of important physiological and pathological processes such as receptor activation, cell growth and differentiation, signal transduction, and immune response by affecting the conformation, localization, stability and folding of proteins. Lewis y antigen is a difucosylated oligosaccharide containing two fucoses, and is a tumor-associated carbohydrate antigen (TACA). Our preliminary study revealed an increase in the expression of Lewis y antigen in approximately 80% of ovarian tumor tissues, which promoted the malignant behavior of ovarian cancer cells including proliferation, adhesion, invasion, metastasis, and drug resistance [1–4]. We also showed that the high expression of Lewis y antigen in ovarian clear cell carcinoma RMG-I-H not only substantially decreased the expression level of cell cycle inhibitor protein p27, but also promoted cells to entry into S phase [5, 6].

p27 is a cyclin-dependent kinase (CDK) inhibitor, which negatively regulates the cell cycle by inhibiting cyclin/CDK activity. During the cell cycle, the expression level of p27 protein is highest in G0/G1 phase, and lowest in S phase. However, the expression level of p27 mRNA displays no significant change throughout the cell cycle. Therefore, the level of intracellular p27 protein depends primarily on its degradation speed instead of the regulation of its expression at the transcription or translation level [7, 8].

Autophagy and the ubiquitin-proteasome system are two main ways of intracellular protein degradation. Autophagy is an evolutionarily conserved physiological process responsible for the degradation of intracellular proteins and damaged organelles in eukaryotes [9]. Intracellular double-membrane-bound-vesicles wrap the cytoplasm, organelles, proteins and other components to form autophagosomes, which fuse with lysosomes to form autolysosomes, and degrade their contents in order to meet the metabolic needs of cells and organelles [10, 11]. The ubiquitin-proteasome pathway consists of two main stages, the ubiquitination of substrate and the degradation process by proteasome. In the classic ubiquitin-proteasome pathway, the ubiquitinated substrate proteins are transported to the 26S proteasome for subsequent degradation [12].

Previous studies have shown that p27 protein is mainly degraded through the ubiquitin-proteasome pathway [13]. However, Yan et al. suggested that the regulatory effect of TGF-β1 on p27 was blocked by the autophagy inhibitor Bafilomycin A1 instead of the proteasome inhibitor MG132, indicating that the expression of p27 may be regulated by autophagy [14]. In this study, we explored the relationship between the expression of Lewis y antigen and autophagy, ubiquitin-proteasome activity, furthermore we investigated whether Lewis y antigen promoted the degradation of p27 through autophagy, the ubiquitin-proteasome pathway or both.

## RESULTS

### High expression of Lewis y antigen promoted the degradation of p27 protein

**Figure 1:**
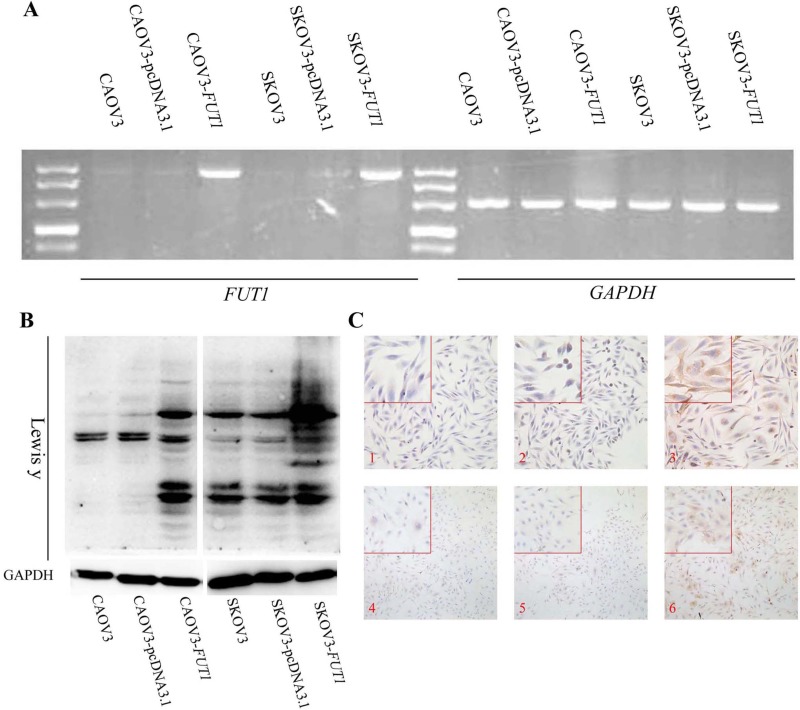
Characterization of FUT1-transfected cell lines (**A**) The expression of *GAPDH* and *FUT1* mRNA in control, the empty vector, and after-transfected cell lines was measured by Reverse transcription PCR. (**B**) The protein level of Lewis y antigen was detected by western blotting in the two groups. (**C**) The immunocytochemical staining method was used to further verify the effect of transfection. 1: CAOV3, 2: CAOV3-pcDNA3.1, 3: CAOV3-*FUT1*, 4: SKOV3, 5: SKOV3-pcDNA3.1, 6: SKOV3-*FUT1*.

To investigate the effect of high expression of Lewis y antigen on cell cycle progression, the CAOV3-*FUT1* and SKOV3-*FUT1* cell lines with high expression of Lewis y antigen were constructed by stable transfection of serous ovarian cancer cell lines CAOV3 and SKOV3 with the α1, 2-fucosyltransferase gene (*FUT1*). The stable transfection of *FUT1* gene into CAOV3 and SKOV3 cell lines was verified at both the gene (Figure 1A) and protein level (Figure 1B and 1C).

**Figure 2:**
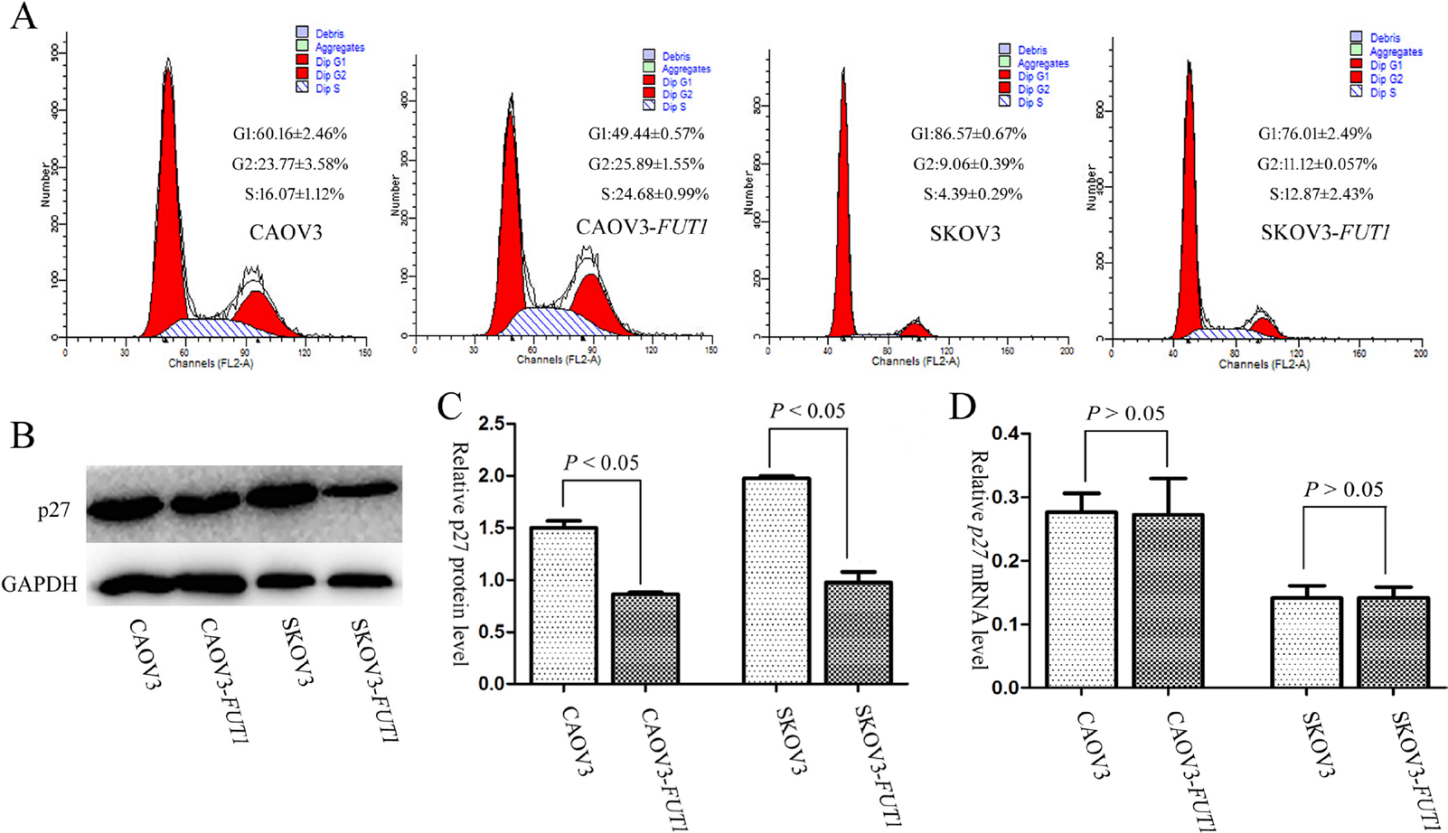
The impacts of Lewis y antigen on cell cycle, the mRNA and protein expression levels of p27 (**A**) Flow cytometry with PI-stained cells showed that in CAOV3-*FUT1* and SKOV3-*FUT1* cells, S phase fractions were increased compared with CAOV3 and SKOV3 cells, respectively. (**B**) The expression of p27 protein in the pre- and post-transfected cell lines was detected by Western blot. (**C**) The quantitative analysis of p27 protein expression in the pre- and post-transfected cell lines. (**D**) The expression of *p27* mRNA in the pre- and post-transfected cell lines was tested by Real-time PCR. Three independent experiments were performed and the results were reproducible.

The changes in cell cycle before and after *FUT1* transfection were compared by flow cytometry. The results suggested that the proportion of S-phase cells increased, but that of G1-phase decreased in the CAOV3-*FUT1* and SKOV3-*FUT1* cell lines, indicating that high expression of Lewis y antigen promoted the progression of cell cycle by stimulating the G1 to S transition (Figure 2A).

Previous studies have shown that p27 is the key regulator in the passage through the restriction point of the G1 to S conversion [15]. We therefore examined the changes in p27 protein expression before and after transfection with the *FUT1* gene, and found out that p27 expression level in CAOV3-*FUT1* and SKOV3-*FUT1* cells was significantly lower than that in CAOV3 and SKOV3 cells (Figure 2B and 2C). Furthermore, we detected the expression of p27 mRNA in the pre- and post-transfected cell lines. Interestingly, there was no significant difference between the pre- and post-transfected cell lines (Figure 2D). The level of intracellular p27 protein depends primarily on its degradation speed instead of the regulation of its expression at the transcription or translation level [7, 8]. So we speculated that Lewis y may regulate p27 protein expression in the post-translational level. Since autophagy and the ubiquitin-proteasome pathway are the two major methods of intracellular protein degradation, we further explored whether the promoted effect of Lewis y antigen on the degradation of p27 was associated with autophagy or ubiquitin-proteasome activity.

### High expression of Lewis y antigen activated autophagy

**Figure 3:**
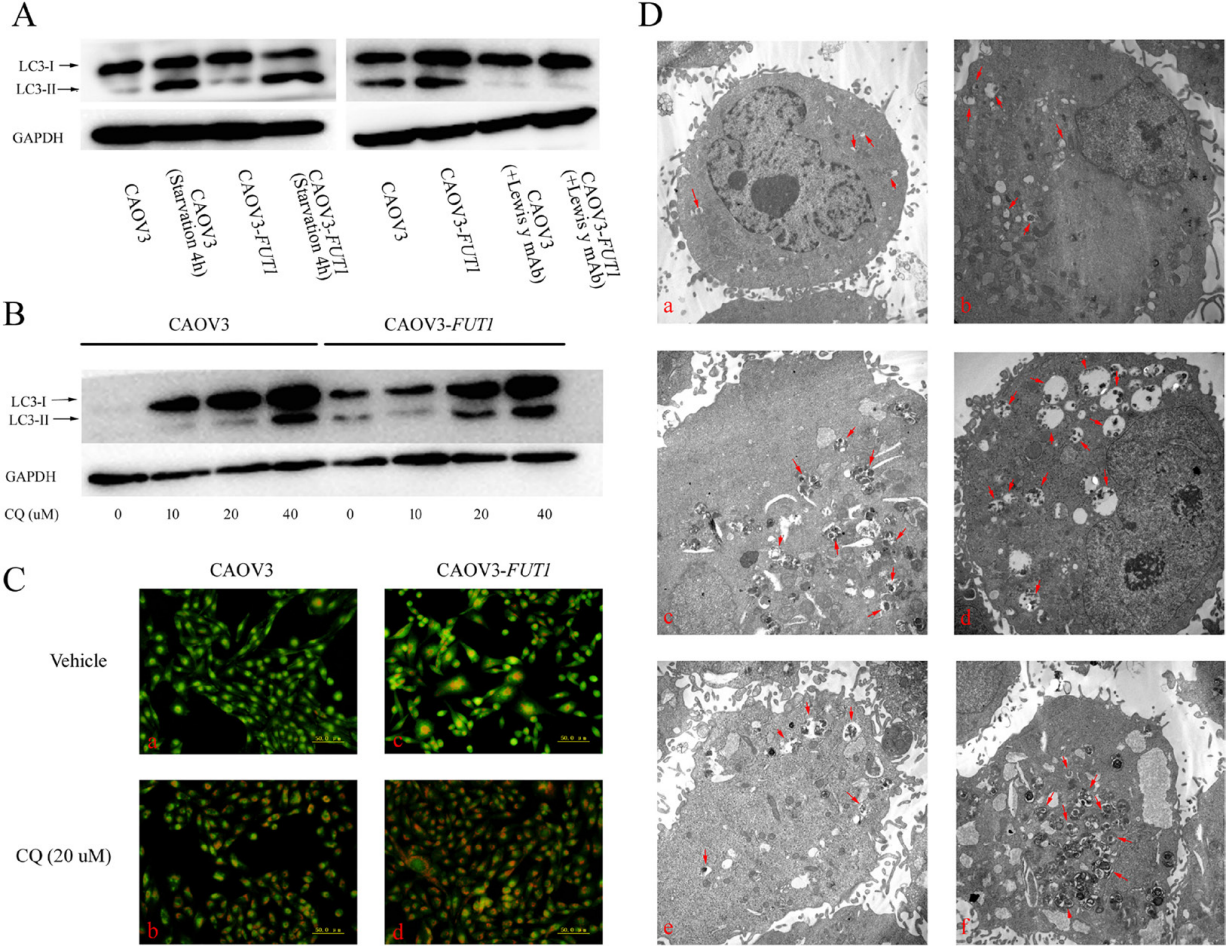
Lewis y antigen overexpression promoted autophagy in the ovarian cancer cell line CAOV3 (**A**) Western blot analysis of LC3-II was performed after exposing the pre- and post-transfected (CAOV3 and CAOV3-*FUT1*) cell lines to different treatments. Cell lines were cultured in normal culture medium, starved for 4 h in EBSS balanced salt solution. Meanwhile, cell lines were cultured in the absence and presence of Lewis y monoclonal antibody. (**B**) CAOV3 and CAOV3-*FUT1* cell lines were incubated with gradient concentrations of the autophagy inhibitor, CQ, for 24 h. The expression of LC3-II was detected by Western blotting. (**C**) CAOV3 and CAOV3-*FUT1* cell lines were treated with vehicle or 20 μM of CQ for 24 h, the formation of acidic vacuoles was analyzed using AO staining. (**D**) Transmission electron microscopy was used to observe the autophagic vacuole number and cellular morphology. a: the CAOV3 cell line was cultured in normal medium; b: the CAOV3-*FUT1* cell line was cultured in normal medium; c: the CAOV3 cell line was starved for 4 h in EBSS balanced salt solution; d: the CAOV3-*FUT1* cell line was starved for 4 h in EBSS balanced salt solution; e: the CAOV3 cell line was treated with 20 μM of CQ for 24 h; f: the CAOV3-*FUT1* cell line was treated with 20 μM of CQ for 24 h.

**Figure 4:**
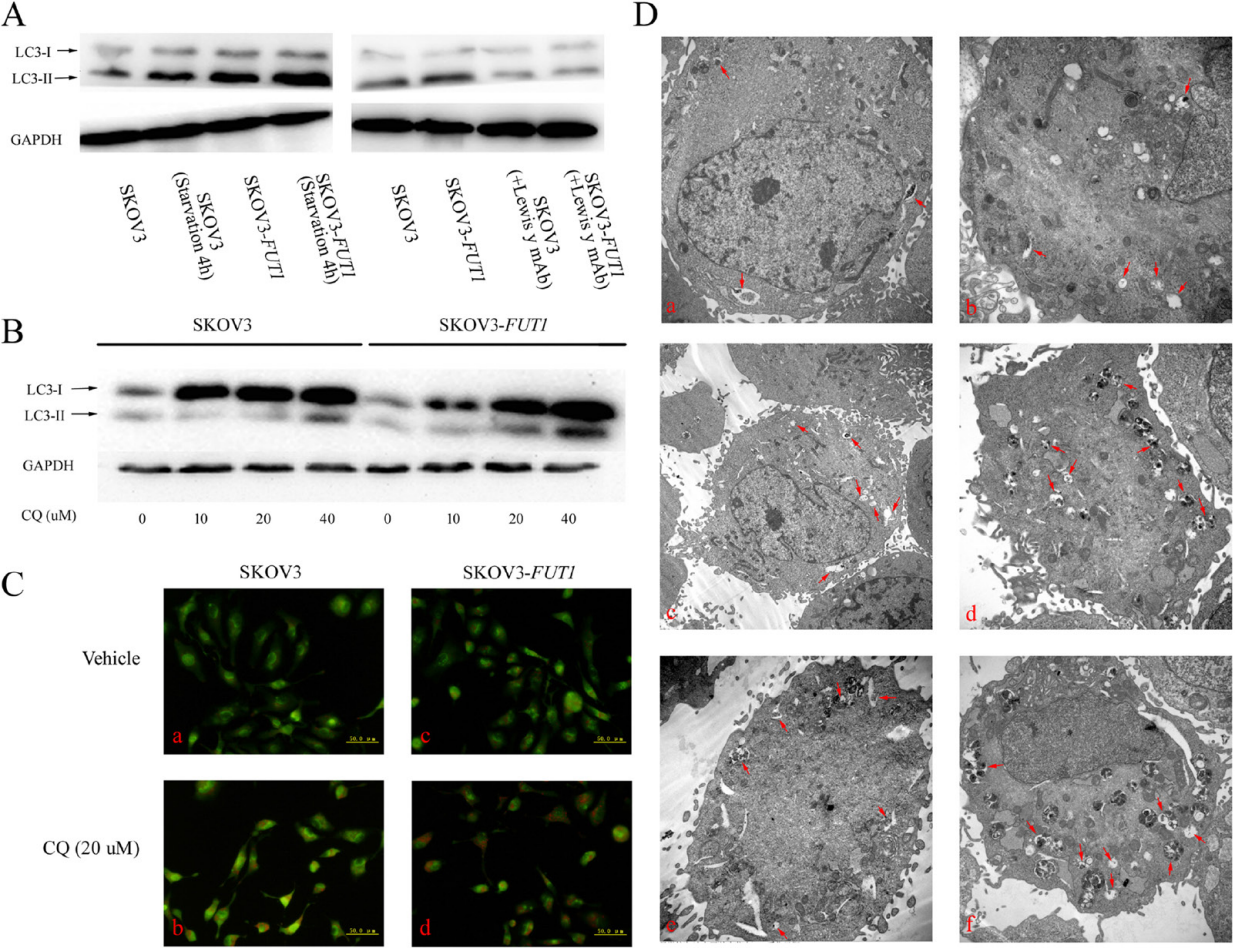
Lewis y antigen overexpression promoted autophagy in the ovarian cancer cell line SKOV3 (**A**) Western blot analysis of LC3-II was performed after exposing the pre- and post-transfected (SKOV3 and SKOV3-*FUT1*) cell lines to different treatments. Cell lines were cultured in normal culture medium, starved for 4 h in EBSS balanced salt solution. Meanwhile, cell lines were cultured in the absence and presence of Lewis y monoclonal antibody. (**B**) SKOV3 and SKOV3-*FUT1* cell lines were incubated with gradient concentrations of the autophagy inhibitor, CQ, for 24 h. The expression of LC3-II was detected by Western blotting. (**C**) SKOV3 and SKOV3-*FUT1* cell lines were treated with vehicle or 20 μM of CQ for 24 h, the formation of acidic vacuoles was analyzed using AO staining. (**D**) Transmission electron microscopy was used to observe the autophagic vacuole number and cellular morphology. a: the SKOV3 cell line was cultured in normal medium; b: the SKOV3-*FUT1* cell line was cultured in normal medium; c: the SKOV3 cell line was starved for 4 h in EBSS balanced salt solution; d: the SKOV3-*FUT1* cell line was starved for 4 h in EBSS balanced salt solution; e: the SKOV3 cell line was treated with 20 μM of CQ for 24 h; f: the SKOV3-*FUT1* cell line was treated with 20 μM of CQ for 24 h.

The microtubule-associated protein 1 light chain 3 (LC3/Atg8) exists in two forms in cells: LC3-I and LC3-II. LC3-I is conjugated to phosphatidylethanolamine to form LC3-II, which is stably retained on the autophagosome membranes until the fusion of autophagosome and lysosome. LC3-II has been widely used as a marker of autophagosomes [16]. As shown in Figure 3A and 4A, although the expression level of LC3-II in both groups was increased 4 h after amino acid deprivation, LC3-II expression was significantly higher regardless at base level or after amino acid deprivation in the post-transfected cells. Furthermore, the LC3-II expression in both groups was significantly decreased 48 h after the cells were treated with Lewis y monoclonal antibody (20 μg/mL), and there was no significant difference in LC3-II expression between the two groups after antibody treatment.

The autophagy inhibitor, chloroquine (CQ), blocks the fusion of autophagosomes and lysosomes by changing the acidic environment of lysosomes, causing the accumulation of ineffective autophagosomes [17]. Autophagic flux refers to the entire dynamic process of autophagosome formation, the transportation of autophagic substrates to lysosomes and lysosomal degradation. In contrast, autophagosomes are only one of the structural components of the autophagy pathway. We therefore measured the intensity of autophagic flux to assess the strength of autophagy activity in cells treated with different concentrations of CQ. It was found that the LC3-II expression in CAOV3-*FUT1* and SKOV3-*FUT1* cells was significantly higher than that in CAOV3 and SKOV3 cells following treatment with gradient concentrations of CQ, although the LC3-II level in all groups was gradually increased with increasing concentrations of CQ (Figures 3B and 4B). These results suggested that Lewis y antigen promoted autophagy flux. We further examined the number of acidic vesicular organelles (AVOs) in cells using acridine orange staining, and found that the staining intensity in untreated and CQ-treated CAOV3-*FUT1* and SKOV3-*FUT1* cells was higher compared with untreated and CQ-treated CAOV3 and SKOV3 cells, respectively (Figures 3C and 4C).

We also examined untreated cells and cells treated with either 4 h of cellular amino acid deprivation or 24 h of CQ (20 μM) in both groups using transmission electron microscopy, the gold standard for the detection of autophagic vacuoles. The number of autophagic vacuoles in CAOV3-*FUT1* and SKOV3-*FUT1* cells was significantly more than that in CAOV3 and SKOV3 cells under these conditions (Figures 3D and 4D).

### High expression of Lewis y antigen promoted the level of ubiquitination in ovarian cancer cells

**Figure 5:**
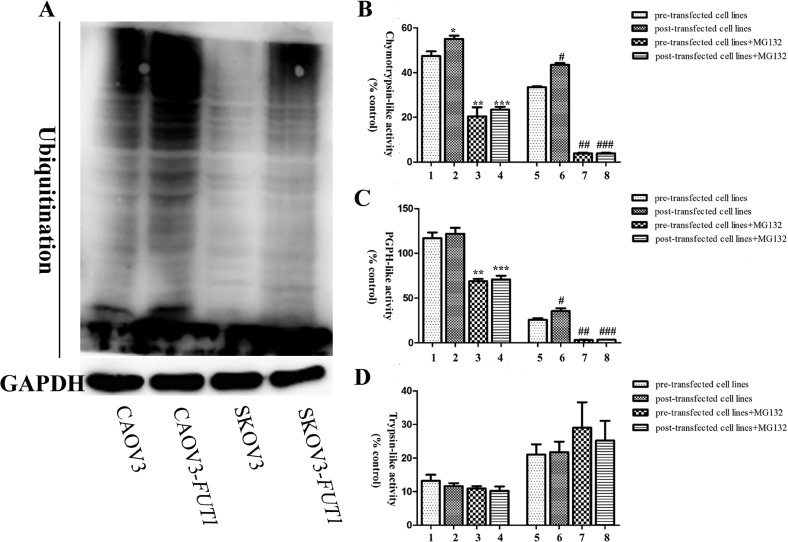
The relationship between the expression of Lewis y antigen and ubiquitin-proteasome system function in the pre- and post-transfected cell lines (**A**) The ubiquitination level in pre- and post-transfected cell lines was detected by Western blotting. (**B**) The pre- and post-transfected cell lines were treated with 20 μM MG132 in DMSO for 24 h. The proteasome extracts were incubated with the fluorogenic proteasome peptide substrate, Suc-Leu-Leu-Val-Tyr-AMC (for the chymotrypsin-like proteasome). (**C**) The proteasome extracts were incubated with the fluorogenic proteasome peptide substrate, Z-Leu-Leu-Glu-AMC (for the PGPH-like proteasome). (**D**) The proteasome extracts were incubated with the fluorogenic proteasome peptide substrate, Z-Val-Val-Arg-AMC (for the trypsin-like proteasome). Following incubation, the hydrolyzed AMCs were quantified using a spectrofluorometer. The values of the error bars are the mean ± SD of three independent experiments. 1: CAOV3; 2: CAOV3-*FUT1*; 3: CAOV3+MG132; 4: CAOV3-*FUT1*+MG132; 5: SKOV3; 6: SKOV3-*FUT1*; 7: SKOV3+MG132; 8: SKOV3-*FUT1*+MG132. ^*^2 compared with 1, *P* < 0.05; ^**^3 compared with 1, *P* < 0.05; ^***^4 compared with 2, *P* < 0.05; ^#^6 compared with 5, *P* < 0.05; ^##^7 compared with 5, *P* < 0.05; ^###^8 compared with 6, *P* < 0.05.

The ubiquitin-proteasome system is an important intracellular proteolytic system that regulates a wide range of biological processes including the cell cycle, cell metabolism, proliferation and differentiation by selective degradation of abnormal proteins and intracellular regulatory proteins. Its abnormal activity could promote tumorigenesis [18]. In order to investigate whether Lewis y antigen promoted the degradation of p27 through the ubiquitin-proteasome pathway, we measured the intracellular level of ubiquitination in both groups, and confirmed the promoted effect of high Lewis y antigen expression on the level of ubiquitination in these cells (Figure 5A).

### High expression of Lewis y antigen promoted the activity of chymotrypsin-like proteasome

Proteasome is the second largest organelle involved in protein degradation after lysosomes. The activities of chymotrypsin-like, trypsin-like, and peptidyl-glutamyl peptide hydrolase-like proteasomes in both groups were detected by the specific fluorogenic substrates, Suc-LLVY-AMC, Z-VVA-AMC and Z-LLG-AMC [19]. The specific amino chains of these substrates were recognized and cleaved by the three types of proteases upon entering the cells, and free illuminophores were released, whose fluorescence intensity was proportional to the activity of the corresponding proteasome. The results demonstrated that chymotrypsin-like proteasome activity in CAOV3-*FUT1* and SKOV3-*FUT1* cells was 16.0% and 29.6% higher than that in CAOV3 and SKOV3 cells, respectively. After the cells were treated with proteasome inhibitor MG132 (20 μM, 24 h), the chymotrypsin-like proteasome activity was markedly decreased in all groups (Figure 5B). The peptidyl-glutamyl peptide hydrolase-like proteasome activity in both CAOV3-*FUT1* and SKOV3-*FUT1* cells was higher than that in CAOV3 and SKOV3 cells, with the most pronounced increase in SKOV3-*FUT1* cells (38.4%) compared with SKOV3 cells. The peptidyl-glutamyl peptide hydrolase-like proteasome activity in all groups was reduced after MG132 treatment (Figure 5C). In addition, there was no significant change in the trypsin-like proteasome activity in CAOV3, CAOV3-*FUT1*, SKOV3, and SKOV3-*FUT1* cells before and after MG132 treatment (Figure 5D).

### Lewis y antigen promoted the degradation of p27 by enhancing the ubiquitin-proteasome activity

**Figure 6:**
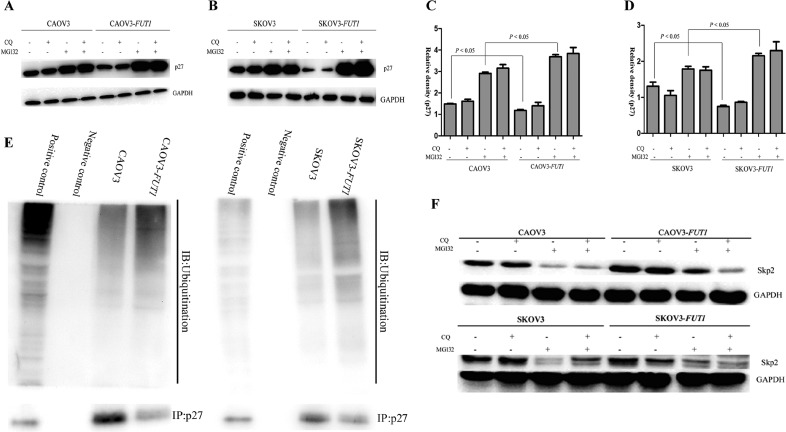
Lewis y antigen promoted the degradation of p27 via the ubiquitin-proteasome pathway, but not autophagy (**A** and **C**) The pre- and post-transfected cell lines were treated with the autophagy inhibitor CQ, proteasome inhibitor MG132, or both. The expression of p27 under different treatments was detected by Western blotting. (**B** and **D**) Quantification of the data from A and C were expressed as the mean ± SD from three independent experiments. (**E**) Immunoprecipitation to detect the ubiquitination of p27. The positive control samples indicated the ubiquitinated level before IP procedure. In the negative control samples, cell lysates were treated the IgG antibody instead of the p27 antibody that precipitated the p27 protein. (**F**) The expression of Skp2 was detected under the cell lines were treated with CQ, MG132, and both by western blotting.

Our studies showed that increased expression of Lewis y antigen promoted autophagy and ubiquitin-proteasome activity. As autophagy and the ubiquitin-proteasome pathway are the two most important pathways in intracellular protein degradation, we investigated whether Lewis y antigen promoted p27 protein degradation by affecting autophagy, ubiquitin-proteasome activity, or both. Cells in all groups were treated with the autophagy inhibitor CQ (20 μM), proteasome inhibitor MG132 (20 μM) and a combination of both for 24 h, then p27 expression was determined by Western blot analysis. As shown in Figure 6A-6D, p27 expression did not change in any of the groups after CQ treatment, whereas p27 expression in all groups was significantly elevated after MG132 treatment, with the highest increase in CAOV3-*FUT1* and SKOV3-*FUT1* cells. These results suggested that the promoted effect of Lewis y antigen on p27 degradation was achieved through the ubiquitin-proteasome pathway. As further proof, the pre- and post-transfected cell lines (contained the positive control and negative control) were simultaneously treated with MG132 50 μg /ml for 24 h, then the cell lysates from indicated cells were immunoprecipitated with anti-p27 and immunoblotted with anti-ubiquitin antibody. The levels of p27 ubiquitination in the CAOV3-*FUT1* and SKOV3-*FUT1* cells were obviously higher than that in the CAOV3 and SKOV3 cells (Figure 6E). This experiment furtherly demonstrated that Lewis y antigen promoted the p27 degradation through the ubiquitin-proteasome pathway. Skp2 was specific enzyme regulating the ubiquitination of p27 [20]. On the purpose of elucidating the possible mechanism that Lewis y antigen affected the ubiquitination of p27, we detected the expression of Skp2 under the cells were treated with CQ, MG132, and both. The expression of Skp2 in the post-transfected cell lines obviously increased compared with pre-transfected cell lines. Skp2 expression did not obviously change in the cell lines after CQ treatment, whereas in all groups was significantly decreased after MG132 treatment (Figure 6F).

### In *vivo* study of the promoted effect of Lewis y antigen on p27 degradation

**Figure 7:**
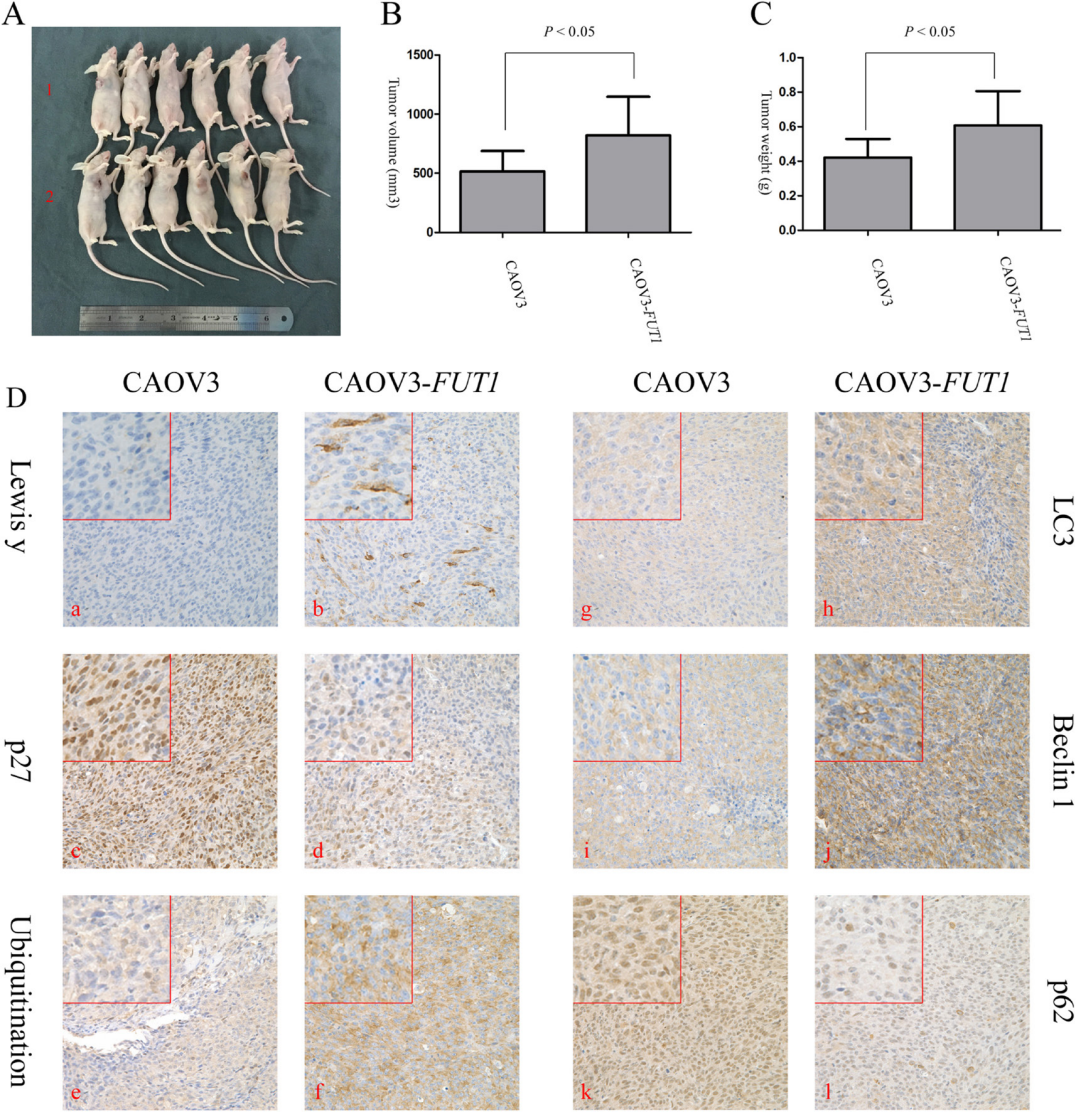
*In vivo* study of the impact of Lewis y antigen on the degradation of p27 (**A**) Photographs show that the xenograft tissues from group 2 were significantly larger than group 1. 1 and 2 represent the CAOV3 group and CAOV3-FUT1 group, respectively. (**B**) Tumor volumes in the two groups were measured with calipers 18 days after subcutaneous injection. Values are presented as means ± SD. (**C**) Tumor weights in the two groups were measured with an electronic balance after the nude mice were humanely killed. Values are presented as means ± SD. (**D**) Immunohistochemical staining of nude mouse xenograft tissues (original magnification, × 400). Lewis y antigen (a and b), p27 (c and d), ubiquitination (e and f), LC3 (g and h), Beclin 1 (i and j), and p62 (k and l).

The impact of Lewis y antigen on the tumorigenic ability was assessed in nude mice injected with pre- and post-transfected cell lines. The body weight of nude mouse in the CAOV3 and CAOV3-*FUT1* groups was: 18 ± 1.83 g and 16.86 ± 2.41 g, respectively, with no significant difference in body weight between these two groups. As shown in Figure 7A–7C, the volume and mass of the transplantable tumor in the CAOV3-*FUT1* group were significantly higher than that in the CAOV3 group. Tumor tissues were then fixed and paraffin-embedded sections were prepared for immunohistochemical examination to compare the expression of Lewis y antigen, p27, the level of ubiquitination, and autophagy-related proteins (LC3, p62 and Beclin 1) in the two groups. Compared with the CAOV3 group, significantly higher expression of Lewis y antigen, higher levels of ubiquitination, LC3 and Beclin 1, but significantly lower expression of p27 and p62 in tumor tissues were observed in the CAOV3-*FUT1* group (Figure 7D). These results further confirmed that Lewis y antigen increased the level of ubiquitination and autophagy activity, and ultimately promoted the degradation of p27 protein.

## DISCUSSION

As an important component of the cell membrane, sugar chains play a crucial role in cell-cell interaction, signal transduction, and adhesion between cells and the extracellular matrix [21]. The structure and the number of sugar chains change during the malignant transformation of cells. In ovarian cancer, these changes mainly occur in the type II sugar chain such as Lewis y antigen. Studies have confirmed high expression of Lewis y antigen in 75% of ovarian cancer tissues, which often indicates poor prognosis in patients [3, 22]. The high expression of Lewis y antigen in the embryonic period and malignant tissues may be associated with its promoted effect on cell proliferation. Our previous study demonstrated that the elevated expression of Lewis y antigen in ovarian clear cell carcinoma cell lines promoted S phase entry by inhibiting p27 expression through the PI3K signaling pathway, and thus promoted cell proliferation [5, 6]. In the present study, progression of the cell cycle was obviously accelerated and p27 expression was significantly reduced in two serous ovarian cancer cell lines CAOV3-*FUT1* and SKOV3-*FUT1*. These results further verified the promoted effect of increased Lewis y antigen expression on the cell proliferation and p27 degradation. During the cell cycle, the expression of p27 protein was highest in G1 phase, and was lowest in S phase. However, the expression level of p27 mRNA shows no significant change throughout the cell cycle, indicating that the level of intracellular p27 protein depends primarily on its degradation speed instead of the regulation of its expression at the transcription or translation level [7, 8]. Autophagy and the ubiquitin-proteasome pathway are the two main pathways of intracellular protein degradation. We therefore speculated that Lewis y antigen may affect the degradation of p27 through autophagy or the ubiquitin-proteasome pathway.

With respect to the effects of the tumor-associated carbohydrate antigen Lewis y on autophagy, our results showed that Lewis y antigen promoted the expression of the autophagic marker, LC3-II, in both untreated cells and starved cells, and this increased expression was blocked by Lewis y monoclonal antibody. Meanwhile, we found that the expression level of LC3-II in all groups was increased when the concentration of CQ increased. Furthermore, the most pronounced increase in LC3-II expression was observed in CAOV3-*FUT1* and SKOV3-*FUT1* cells, indicating that Lewis y antigen promoted autophagic flux in these cells. The promoted effect of Lewis y antigen was further confirmed by AO staining and transmission electron microscopy. Lewis y antigen is a structural component of various glycoprotein receptors on the cell surface such as integrin β1 and EGFR. Our preliminary results demonstrated that integrin β1 and EGFR as well as their relative content of Lewis y antigen in cells transfected with the *FUT1* gene were significantly increased [1, 5]. There are currently few reports on the correlation between integrins and autophagy, and this association remains controversial. In lung adenocarcinoma cell lines, autophagy can be promoted by inhibiting the activity of integrin β4 [23]. However, Edick MJ et al. showed that integrin α3β1 plays an important role in the induction of autophagy in prostate epithelial cells [24]. Barry Jutten et al. demonstrated that cells overexpressing EGFR are more sensitive to the therapy with the autophagy inhibitor CQ [25]. Chen et al. also found that cathepsin S induced autophagy through the EGFR-ERK signaling pathway [26]. We therefore speculate that increased Lewis y antigen expression may activate a series of signaling pathways downstream of the receptor by changing the conformation of integrin β1 and EGFR, affect the level of autophagy in ovarian cancer cells, and ultimately promote the progression of malignant tumor.

As the most important pathway of intracellular protein degradation in addition to autophagy, the ubiquitin-proteasome system plays a vital role in the regulation of cell cycle and tumor growth, and has become a target for the treatment of malignant tumors [27]. Bazzaro M et al, have shown that the levels of polyubiquitinated proteins were 2- to 3-fold higher in malignant tumors compared with serous cystadenoma, meanwhile, when the proliferation rate of ovarian cancer cell lines decreased, the polyubiquitinated protein levels significantly decreased [28]. In this study, we confirmed that Lewis y antigen overexpression promoted the level of ubiquitination in ovarian cancer cells. As shown in our previous published papers, we have demonstrated that Lewis y antigen promoted the proliferation of ovarian cancer cells [29, 30]. The increased ubiquitination level may be correlated with the elevated proliferation rate and higher malignant degree in the post-transfected cell lines. We further determined the activities of chymotrypsin-like, peptidyl-glutamyl peptide hydrolase-like, and trypsin-like proteasomes and revealed a significant increase in chymotrypsin-like proteasome activity in both groups. Moreover, MG132 treatment significantly reduced the activity of chymotrypsin-like. Young Hwang et al. showed that MG132 can significantly inhibit the chymotrypsin-like proteasome activity in several ovarian cancer cell lines [31]. The intracellular chymotrypsin-like proteasome activity has been proven to be closely related to the state of tumor cells [32, 33]. Our results further confirmed that Lewis y antigen may promote the proliferation and survival of ovarian cancer cells by increasing intracellular chymotrypsin-like proteasome activity.

Ubiquitin-proteasome mediated protein degradation is an important regulatory mechanism in cell cycle progression. The process of p27 degradation by the ubiquitin-proteasome system has been extensively studied. p27 protein is linked to the ubiquitin following recognition by the specific E3 ubiquitin ligase -Skp2, and enters proteasomes for degradation [20, 34]. In recent years, the number of studies on p27 protein and autophagy has gradually increased. Liang et al. showed that p27 Thr198 phosphorylation regulated cell cycle and macroautophagy [35]. Ding et al. suggested that p27 degradation might be mediated by the autophagy pathway [14]. In the present study, in order to investigate the specific way of Lewis y antigen in p27 degradation, we compared p27 expression in cells treated with the autophagy inhibitor CQ, proteasome inhibitor MG132, or a combination of both. The results suggested that p27 expression in CQ-treated cells was unchanged, whereas the expression in MG132-treated cells was significantly increased. In a study by Bazzaro M et al., MG132 increased the intracellular level of p27 and induced apoptosis by activating caspase-3 in the ovarian cell line ES-2 [28]. Consistent with their findings, the level of p27 protein in CAOV3-*FUT1* and SKOV3-*FUT1* cells was significantly increased after MG132 treatment, which may be related to the more pronounced inhibitory effect of MG132 due to higher chymotrypsin-like proteasome activity in these cells. What's more, after the proteasome activity was blocked by MG132, the levels of p27 ubiquitination in the post-transfected cells were significantly higher than that in the pre-transfected cells. Meanwhile, the expression of Skp2 increased in the post-transfected cells, and significantly decreased after MG132 treatment in any of the groups. These experiments may furtherly confirm that Lewis y antigen promoted the degradation of p27 via enhancing the ubiquitin-proteasome activity.

This is the first study to investigate the relationship between Lewis y antigen, autophagy and the ubiquitin-proteasome system. Lewis y antigen promotes the degradation of p27 protein by stimulating the ubiquitin-proteasome system, accelerating the cell cycle and proliferation, and ultimately promotes malignant behaviors in ovarian cancer cells.

## MATERIALS AND METHODS

### Chemical reagents

RPMI-1640 medium and fetal bovine serum (FBS) were obtained from Gibco (Rockville, MD, USA). Lipofectamine^®^ LTX with Plus^TM^ Reagent and Acridine Orange were purchased from Invitrogen Life Technologies (Carlsbad, CA, USA). The PrimeScript^TM^ 1st Strand cDNA Synthesis kit, PCR Amplification kit and Real-time PCR kit were from Taraka (Dalian, Liaoning, China). Antibodies against Lewis y antigen, p27, Beclin 1, and GAPDH were from Abcam (Cambridge, UK). Antibodies for ubiquitin, microtubule associated protein 1 light chain 3 (LC3), Skp2 and p62 were purchased from Cell Signaling Technology (Beverly, MA, USA). Propidium iodide (PI), RNase, and Chloroquine (CQ) were from Sigma-Aldrich (St. Louis, MO, USA). MG-132 was from Calbiochem (San Diego, CA, USA). Fluorogenic peptide substrates Suc-Leu-Leu-Val-Tyr-AMC (for the proteasomal chymotrypsin-like activity), Z-Val-Val-Arg-AMC (for the proteasomal trypsin-like activity), and Z-Leu-Leu-Glu-AMC (for the proteasomal peptidylglutamyl peptide hydrolasel-like, PGPH-L activity) were obtained from Sangon Biotech (Shanghai, China).

### Cell lines and cell culture

The CAOV3, SKOV3 cell lines were obtained from American Type Culture Collection. All cells were cultured in RPMI 1640 medium supplemented with 10% fetal calf serum. The cells were maintained in a 5% CO_2_ atmosphere at 37°C. The human α1, 2-fucosyltransferase gene, *FUT1*, was amplified by PCR with genomic DNA from human leukocytes as a template and was transfected into CAOV3 and SKOV3 cells. The plasmid was transfected with Lipofectamine^®^ LTX with Plus^TM^ Reagent following the manufacturer's protocols. The stable transfectants were named CAOV3-*FUT1* and SKOV3-*FUT1* and they were selected in the presence of G418 (800 μg/mL for CAOV3, 400 μg/mL for SKOV3). These cell lines grew in monolayers and were passaged when cultures were 70–80% confluent.

### Cell-cycle analysis by flow cytometry

Monodispersed cells (1 × 10^6^) were harvested during the exponential growth phase. The cells were washed with phosphate buffered saline (PBS), fixed in 75% ethanol, and stored at –20°C overnight and then washed twice in PBS. The cells were then re-suspended in 500 μL of PBS and stained with a 200 μL solution containing propidium iodide (50 μg/mL) and RNase (20 μg/mL) and protected from light for 30 min. The distribution of cells in the cell cycle was analyzed using a flow cytometer (BD Company, Franklin Lakes, NJ, USA). All experiments were performed at least three times.

### Immunocytochemistry and Immunohistochemistry

The cells were seeded on coverslips and fixed by 4% of paraformaldehyde, then stained according to the SABC test kit instructions. In brief, after blocking with goat serum for 1 h at 37°C, mouse anti-human Lewis y antigen antibody (1:100) was added and the slides were incubated overnight at 4°C. Lewis y antigen immunostaining was performed using the avidin-biotin peroxidase complex kit and then photographed. The presence of brownish yellow granules in the cytoplasm and cell membrane was considered a positive result.

Paraffin-embedded histological samples of tumor xenografts were cut into 5 μm sections. The specific method referred to previous literature [36]. The working concentrations of primary antibodies against Lewis y antigen, p27, ubiquitination, LC3, Beclin 1, and p62 were 1:50, 1:200, 1:400, 1:200, 1:200, and 1:200, respectively.

### Reverse transcription PCR (RT-PCR) and Real-time PCR

Total RNA from cell lines was isolated using TRIzol^®^ LS Reagent (Invitrogen, Carlsbad, CA, USA). Briefly, the experimental methods for Reverse-Transcription and Real-time PCR were respectively performed as previous described [36, 37]. The primer sets used for the Reverse transcription PCR and Real-time PCR were listed in Table 1.

**Table 1:** The primer sequences used in this study

Gene Name	Primer Sequence	Products (bp)
*FUT1*	F:5′- ATGTGGCTCCGGAGCCATCGTCAG-3′	480
(Reverse-Transcription PCR)	R:5′- AGGATCTCTCAAGTCCGCGTACTC-3′	
*GAPDH*	F:5′-AGGAGCGAGATCCCTCCAAA-3′	328
(Reverse-Transcription PCR)	R:5′- GTCTTCTGGGTGGCAGTGAT-3′	
*p27*	F:5′- CAAATGCCGGTTCTGTGGAG -3′	177
(Real-time PCR)	R:5′- TCCATTCCATGAAGTCAGCGATA -3′	
GAPDH	F:5′- AAGGCTGTGGGCAAGG -3′	238
(Real-time PCR)	R:5′- TGGAGGAGTGGGTGTCG -3′	

### Immunoprecipitation and immunoblotting

After the cells were cultured with different treatment factors, cells were collected, washed twice with cold PBS, and lysed in cell lysis RIPA Buffer for 30 min on ice. The immunoprecipitation and immunoblotting were performed as previously described [38].

### Peptidase activities of the proteasome and the whole cell extracts

After the cells were cultured and treated with 20 μmol/L MG-132 for 24 h at 37°C in six-well plates, the cells were thoroughly scraped from the culture dishes with a cell scraper and washed with cold PBS. Proteasomes were extracted according to previous description [39, 40]. Peptidase activities of the proteasome were determined by measuring hydrolysis of the fluorogenic substrates, Suc-Leu-Leu-Val-Tyr-AMC, Z-Val-Val-Arg-AMC, and Z-Leu-Leu-Glu-AMC. These substrates are preferentially hydrolyzed by the chymotrypsin-like, the trypsin-like and the peptidyl glutamyl peptide hydrolase (PGPH)-like activities of the proteasome, respectively. Ten microliters (1 μg/μL) of each freshly made supernatant was incubated in a 96-well plate at 37°C for 30 min with 10 μL of 250 μmol/L Suc-Leu-Leu-Val-Tyr-AMC, 500 μmol/L Z-Val-Val-Arg-AMC, 500 μmol/L Z-Leu-Leu-Glu-AMC and 85 μL assay buffer (20 mmol/L Tris-HCl, pH 7.5, and 10% glycerol). DMSO (10 μL) instead of fluorogenic substrate was incubated with the proteasome supernatant and was used as the negative control. The release of fluorescent AMC was measured with a spectrofluorometer (Perkin-Elmer Life and Analytical Sciences, Inc., Wellesley, MA, USA) at 460 nm with an excitation wavelength of 380 nm.

### Acridine orange staining for acidic vesicular organelles

Acridine orange was added at a final concentration of 1 μg/mL for 15 min, and then washed with PBS. Images were obtained using a fluorescence microscope (Olympus) equipped with a digital camera (Olympus).

### Transmission electron microscopy

All cells with different treatments were harvested using 0.25% trypsin, washed with cold PBS and collected by centrifugation for 15 min at 1500×g. Then, the following operation referred to previous description [41].

### *In vivo* treatment

Female BALB/c nu/nu mice (4–6-weeks-old; Beijing HFK Bioscience Co., LTD) were raised in specific pathogen-free conditions. The experimental protocol was approved by the Institutional Review Board of Shengjing Hospital of China Medical University and the ethic approval code was “2015PS169K”. Twelve mice were randomly divided into 2 groups and were respectively injected with the pre- and post-transfected ovarian cancer cell lines (CAOV3 and CAOV3-*FUT1*). Cells in the exponential phase of growth were digested by 0.25% trypsin and re-suspended in PBS. A volume of 0.2 mL (5 × 10^6^ cells) was respectively injected into the bilateral upper flank region. The mice were then killed humanely 18 days after subcutaneous injection and an autopsy was performed. Tumor growth was monitored by measuring 2 bisecting diameters of each tumor using a caliper every 3 days. The tumor volume was calculated using the formula (V = a × b^2^/2), where a represents the largest diameter and b the smallest diameter. The tissues were then dehydrated, processed, and embedded in paraffin wax. Serial sections 5 μm thick were prepared from immunohistochemistry.

### Statistical analysis

All data were expressed as the mean ± SD of three separate experiments. Statistical comparisons were made using the Student's *t-*test. *P*-values of less than 0.05 were considered statistically significant.
